# Lipids and Lipoproteins in Atherosclerosis

**DOI:** 10.1155/2011/160104

**Published:** 2012-03-20

**Authors:** Angeliki Chroni, George Leondaritis, Helen Karlsson

**Affiliations:** ^1^Institute of Biology, National Center for Scientific Research “Demokritos”, Agia Paraskevi, 15310 Athens, Greece; ^2^Biomedical Research Foundation of the Academy of Athens and Department of Pharmacology, Medical School, University of Thessaly, 41110 Larissa, Greece; ^3^Department of Occupational and Environmental Medicine, Linköping University Hospital, 58185 Linköping, Sweden

Atherosclerosis is a focal disease of the arterial wall that leads to cardiovascular disease (CVD), the biggest cause of morbidity and mortality in Western societies. Atherosclerosis is a complex, chronic, progressive disease that affects large and medium-sized arteries. Atherosclerotic lesions are promoted by low-density lipoproteins and form from accumulation of fatty substances, cholesterol, cellular waste products, calcium, and fibrin in the inner lining of the arterial wall. Lipoproteins are complexes of amphipathic proteins with lipids at variable ratios, densities, and sizes. Their role is to transport water-insoluble lipids in the blood. Plasma lipoproteins have traditionally been grouped into five major classes, based on their buoyant density: chylomicrons, very low-density lipoproteins (VLDL), intermediate-density lipoproteins (IDL), low-density lipoproteins (LDL), and high-density lipoproteins (HDL). It is believed that atherogenic lipoproteins, such as LDL and lipoprotein remnants, that float in the VLDL IDL region, promote atherosclerosis, and antiatherogenic lipoproteins, such as HDL, protect from atherosclerosis. Despite many advances in cardiology, atherosclerosis remains an important medical problem suggesting that some steps in pathogenic mechanisms remain unclear.

This special issue contains a series of reviews and original research articles that seek to provide insight into the role of lipids and lipoproteins in health and disease with emphasis given on their implication in atherosclerosis. The first review by H. Itabe et al. describes the *in vivo* dynamics of oxidized LDL during atherosclerosis, and the second one by O. Tehlivets discusses the link of homocysteine, dysregulated lipid metabolism, and atherosclerosis. Following these reviews new data on the prediction and pathogenesis of atherosclerosis are presented in four research papers. M. Enomoto et al. investigate the issue of the best predictor of subclinical atherosclerosis and provide suggestions that the LDL-cholesterol/HDL-cholesterol ratio is a better predictor of carotid intima-media thickness progression than HDL-cholesterol or LDL-cholesterol alone. J.-B. Hansen et al. suggest that the content of apoC-I per VLDL particle is an important regulator of triglyceride metabolism in the fasting and postprandial state and associated with carotid atherosclerosis. M. Guha and O. Gursky show that human plasma VLDL are destabilized by acidic pH. They propose that the acidic environment found in the advanced atherosclerotic plaques could destabilize VLDL, enhancing their fusion and coalescence into lipid droplets, such as the droplets found in atherosclerotic plaques. J. Oestvang et al. suggest that lysophosphatidylcholine and platelet activating factor stimulate arachidonic acid release by divergent pathways in monocytes. Elucidation of monocyte lysoPC-specific signaling mechanisms will aid in development of novel strategies for atherosclerosis prevention, diagnosis, and therapy. Then two papers (one review and one research paper) deal with the association of lipid abnormalities with common diseases and predisposition to CVD. M. Peppa et al. discuss the observed dyslipidemia and increased risk of coronary artery disease, cerebral ischemia, and angina pectoris in older and possibly ischemic stroke in younger patients with overt or subclinical hyperthyroidism. P. M. Rocha et al. provide data that reinforce the contribution of an abdominal obesity phenotype associated with a diabetogenic and atherothrombotic profile to liver lipotoxicity.

We hope that this collective special issue will help to update and enrich current research on atherosclerosis and CVD.

*Angeliki Chroni* *Angeliki Chroni* *George Leondaritis* *George Leondaritis* *Helen Karlsson* *Helen Karlsson*

